# Differences in Healing of a Horizontal Root Fracture as Seen on Conventional Periapical Radiography and Cone-Beam Computed Tomography

**DOI:** 10.1155/2017/2728964

**Published:** 2017-07-04

**Authors:** Ronnachat Rothom, Patchanee Chuveera

**Affiliations:** ^1^Dental Department, Hangchat Hospital, Lampang, Thailand; ^2^Department of Family and Community Dentistry, General Dentistry Branch, Faculty of Dentistry, Chiang Mai University, Chiang Mai, Thailand

## Abstract

Different locations and healing patterns of horizontal root fractures bear different prognoses. Conventional periapical radiographs have been routinely used for the evaluation of the locations and healing of horizontal root fractures, with the limitation of presenting two-dimensional images. The three-dimensional imaging technology, cone-beam computed tomography (CBCT), has recently gained interest in dental traumatology, in particular for locating and diagnosing root fractures. However, the assessment of healing patterns of horizontal root fracture using CBCT compared to conventional radiographs has not been established. This case report describes the different healing patterns evaluated by two-dimensional radiographs and CBCT of a horizontally root-fractured maxillary right central incisor treated with mineral trioxide aggregate (MTA) with a two-year follow-up. The findings suggest that the healing patterns of horizontal root fractures seen on conventional radiographs and CBCT may be different.

## 1. Introduction

Horizontal root fractures are uncommon, accounting for 0.5–7% of all injuries to permanent teeth [1]. Teeth with horizontal root fractures often present with mobility, extrusion, and displacement of the coronal fragment in varying degrees depending on the location of the fracture and the severity of trauma [2].

The definitive diagnosis of horizontal root fracture needs a radiographic assessment. Since the fracture may not be detected if the central X-ray beam does not pass directly through the narrow diastasis [3, 4]. Additional radiographs with increased or decreased vertical angulation of 15 degrees are specifically suggested for diagnosis of horizontal root fractures [1]. Recently, cone-beam computed tomography (CBCT) has been recommended as the imaging modality of choice for diagnosis and management of dentoalveolar trauma, including root fractures [3, 5]. The advantage of CBCT technology is that it provides three-dimensional visualization of anatomic structures. It is noted that it may be useful for detecting the presence, exact location, extent, direction, and angulation of the fracture without superimposition of other structures [3]. However, CBCT has some limitations, such as limited availability, high cost, and high level of radiation [3, 5, 6].

The location and healing classification of horizontal root fractures are important factors in determining prognosis [7]. However, clinical studies evaluating location and healing of horizontal root fractures commonly use conventional radiographic assessment [2, 8, 9]. Studies comparing location and healing types classified by CBCT and conventional radiographs are lacking [10, 11].

The purpose of the present case report is to describe the clinical outcomes and radiographic findings from two-dimensional radiographs and CBCT in a two-year follow-up of a maxillary right central incisor with a horizontal root fracture when only the coronal fragment was treated with mineral trioxide aggregate (MTA).

## 2. Case Report

**Figure 1:**
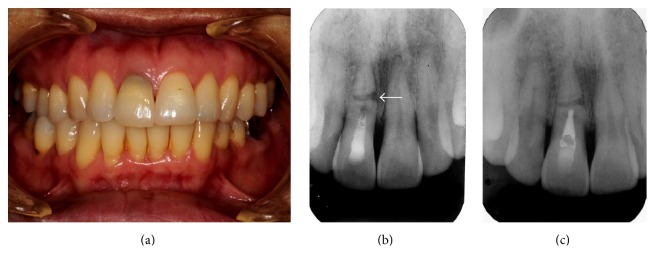
(a) Initial intraoral photograph (June 2012) reveals tooth discoloration and slight extrusion of tooth 11. (b) Initial periapical radiograph of tooth 11 (June 2012) reveals a middle-third horizontal root fracture and underfilled root canal medication. Discontinuation of lamina dura with a radiolucent area is noticed on the mesial aspect of the fracture site (arrow). (c) The periapical radiograph one year after medication with Vitapex (June 2013) shows improved healing. A continuous lamina dura is seen on both sides of the fracture and the apical fragment appears within normal limits.

A 48-year-old Thai woman came to the Faculty of Dentistry, Chiang Mai University, in March 2012, with a chief complaint of spontaneous pain and mobility in tooth 11 resulting from a fall accident four months earlier. Tooth 11 was diagnosed with a horizontal root fracture and pulp necrosis with symptomatic apical periodontitis of coronal fragment. Emergency treatment was provided in the form of preliminary cleaning of the root canal of the coronal fragment and placing calcium hydroxide medication without tooth splinting. Definitive root canal treatment was scheduled two months later. Baseline intraoral photograph showing tooth discoloration (Figure 1(a)) and periapical radiographs (Figure 1(b)) were made prior to the initiation of definitive root canal treatment. A calcium hydroxide paste, Vitapex (Neo Dental Chemicals, Tokyo, Japan), was used as an intracanal medication in the coronal fragment. The apical fragment was left untreated.

**Figure 2:**
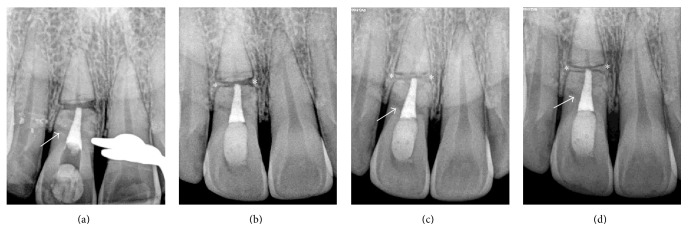
Digital periapical radiographs. (a) Radiograph immediately after MTA obturation in coronal root fragment (June 2013) illustrates approximately 0.5 mm extruded MTA at the apical end of the coronal fragment and a suspected fracture line at the level of the cervical third on the distal aspect of the root (arrow). (b) Radiograph one year after MTA obturation (June 2014) reveals the ingression of bone into the diastasis from both mesial and distal aspects of the fracture, but it does not fill the central aspect of the diastasis (asterisks). The apical root fragment has a continuous lamina dura without apical radiolucency. ((c) and (d)) Radiographs two years after MTA obturation (June 2015) show complete healing at the diastasis (asterisks). A radiolucent line at the distal aspect of the cervical third of the root is still noticed (arrows).

**Figure 3:**
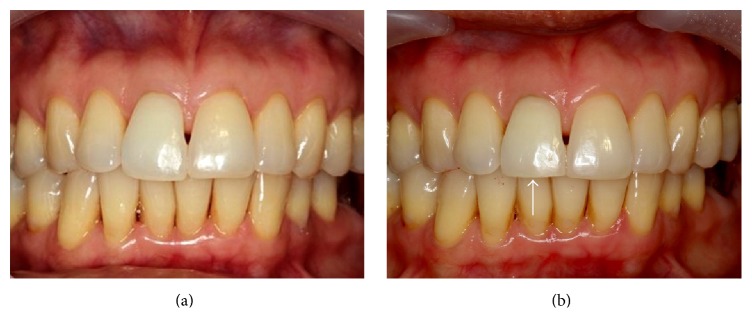
(a) Intraoral photograph after three cycles of internal bleaching of tooth 11 (July 2013) shows that the color was brighter than that of adjacent teeth (b) The incisal edge of tooth 11 was slightly reduced to minimize loading (arrow) (June 2015). The color of tooth 11 has not changed when compared with the tooth color immediately after MTA obturation and intracoronal bleaching performed two years previously.

One year after calcium hydroxide medication, the tooth was asymptomatic. A periapical radiograph showed resolution of the lateral radiolucency with a continuous lamina dura on both sides of the fracture and the apical fragment appeared to be within normal limits (Figure 1(c)). The root canal of the coronal fragment was then obturated with MTA (ProRoot MTA, Dentsply-Maillefer, Ballaigues, Switzerland). A digital periapical radiograph was made immediately after MTA obturation (Figure 2(a)). One month later, intracoronal bleaching was performed using glass ionomer restorative material (GC Fuji IX™ GP, GC America, Alsip, IL, USA) as a cervical barrier and sodium perborate mixed with water as bleaching agent. After three cycles of bleaching, the color was brighter than that of adjacent teeth (Figure 3(a)). The access cavity was then restored with resin composite (Fitek Z350 XT, 3M ESPE, St. Paul, MN, USA).

One year after MTA obturation, the tooth was asymptomatic and the patient was satisfied with the color. A periapical radiograph (Figure 2(b)) revealed the ingression of bone into the diastasis from both mesial and distal aspects of the fracture but did not fill its central aspect. The apical root fragment had a continuous lamina dura without apical radiolucency.

**Figure 4:**
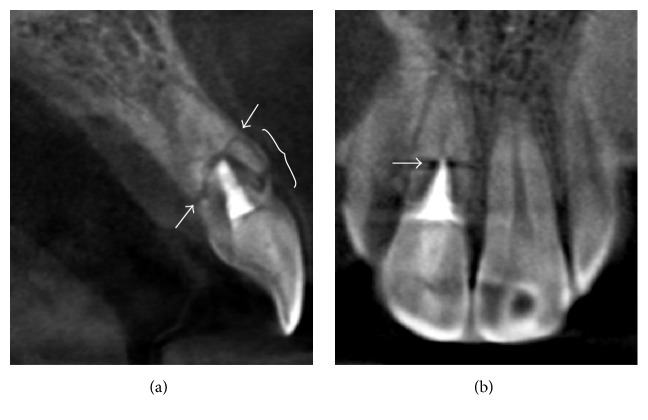
CBCT images (June 2015). (a) The sagittal CBCT slice of tooth 11 reveals a comminuted fracture on the labial aspect within the cervical third of the root (brace). A complete fracture line was found running obliquely from the middle third on the facial aspect through the cervical third on the palatal aspect (arrows). (b) The coronal CBCT slice reveals a horizontal root fracture in the middle third of the root without interposition of hard tissue (arrow).

At two-year follow-up (June, 2015), two periapical radiographs were made, one with the paralleling technique (Figure 2(c)) and the other with a decreased vertical angulation of the X-ray beam (Figure 2(d)). Both periapical radiographs showed improved healing at the diastasis. A distinct radiopaque line between the coronal and apical fragments made each fragment look as if it was separated by bone and surrounded by its own lamina dura. Based on these findings, the healing was classified as “healing by interposition of bone and connective tissue” according to the definition established by Andreasen and Hjörting-Hansen [8]. However, another radiolucent line was noticed at the distal aspect of the cervical third (Figures 2(c) and 2(d)). An additional cervical fracture was suspected but that diagnosis was inconclusive since there was no clinical sign of increased tooth mobility. To rule out additional fracture, CBCT was prescribed (Orthophos XG 3D®, Sirona, Bensheim, Germany). A sagittal CBCT slice (Figure 4(a)) revealed an unexpected comminuted fracture on the labial aspect within the cervical third of the root. A complete fracture line was found running obliquely from the middle third on the facial aspect through the cervical third on the palatal aspect. There was no ingression of hard tissue into the diastasis of the complete oblique fracture seen on CBCT. For the apical fragment, no periapical lesion was found. A coronal CBCT slice (Figure 4(b)) revealed a horizontal root fracture in the middle third of the root without interposition of hard tissue at the diastasis. Healing by interposition of connective tissue was suspected instead, based on the CBCT findings.

Since a comminuted fracture was revealed in addition to the previously detected horizontal root fracture, the incisal contacts were carefully checked and reduced to minimize loading during functional movement. It was also noticed that the color of tooth 11 was stable when compared with the color after intracoronal bleaching which was performed two years previously (Figure 3(b)).

Written informed consent for the case to be published was obtained from the patient for publication of this case report.

## 3. Discussion

A study by Bornstein et al. [12] compared the use of CBCT and intraoral radiographs in assessing the location in the same subjects. The authors found that the location and angulation of the fractures, based on CBCT and intraoral radiographs, were significantly different. The most common location detected by intraoral radiographs was the middle third (63.6%). With CBCT, middle third root fracture occurred 70.45% on the facial aspect and 29.55% on the palatal aspect of the root. It can be inferred that transverse middle third fractures seen in periapical radiographs are actually oblique, with middle third fractures on the facial aspect and cervical third fractures on the palatal aspect in most cases. The location of the main horizontal fracture in our case report, based on conventional periapical radiographs, was in the middle third of the root, while, based on a sagittal CBCT image, it was oblique. One end lied in the middle third on the facial aspect and the other in the cervical third on the palatal aspect. Hence, the findings in our case are concordant with those of Bornstein et al. [12].

The location of the facture is an important factor affecting the prognosis and long-term survival of the tooth [2, 7, 13]. A study by Andreasen and cowokers [7] found that the 10-year survival rate of horizontal root fractures at the apical third level was 89%, at the middle third 78%, at the cervical-middle 67%, and at the cervical third 33%. It is assumed that the 10-year survival rate in our case report, based on CBCT, would be between 33% and 67%, which is less than the prediction made by conventional radiographs.

Based on conventional radiographic studies, the most common type of healing was interposition of connective tissue, followed by healing with calcified tissue and interposition of granulation tissue, whereas the least common type was interposition of bone and connective tissue [1, 2, 9]. Andreasen and coworkers [7] found that, in connective tissue healing, the survival rate was decreased by the location of the fracture but in calcified tissue healing, there was no tooth loss after eight years' follow-up, regardless of the position of the fracture. The types of healing assessed by conventional periapical radiographs and CBCT in our case were different. However, considering our patient's age, the healing seen in conventional periapical radiographs, interposition of bone and connective tissue, was uncommon [1]. The healing evaluated by CBCT in this case, which was classified as interposition of connective tissue, may be more accurate.

Hence, the long-term survival and prognosis of this tooth, based on CBCT, were considered worse than that expected from conventional radiographs. However, the additional comminuted fracture and different healing classification in our case did not result in explicitly increased tooth mobility or other clinical findings suggestive of failure at two-year follow-up.

Generally, endodontic treatment immediately after horizontal root fracture is not recommended [8]. Root canal treatment is initiated only when pulp necrosis is confirmed [6]. It has been observed that pulp necrosis in horizontal root fracture occurs in 20–40% of cases [1] and when pulp necrosis develops, the apical fragment usually remains vital [8]. According to this finding, root canal treatment is recommended in the coronal fragment only.

The use of MTA as a total obturation material in the coronal root fragment is practical because the coronal root canal fragment is usually quite short and needs adequate MTA thickness to seal [9, 14–16]. A recent retrospective study evaluated healing of horizontal root fractures treated with MTA at three-year average follow-up periods and found a highly satisfactory healing outcome of 89.5% [9]. This case report supports the principal of treating with MTA only the coronal fragment in horizontal root fracture since there was no pathological change in the apical root fragment or secondary inflammation around the comminuted fracture detected by CBCT at follow-up.

Weakening of radicular dentin after long-term exposure to calcium hydroxide has been reported [17–19]. A systematic review by Yassen and Platt [19] concluded that exposure to calcium hydroxide for five weeks or longer could reduce the mechanical properties of radicular dentin. Therefore, the comminuted fracture found on the labial surface of the coronal root fragment in our case might have occurred later because of long-term medication with calcium hydroxide.

May et al. [3] suggested using CBCT when (1) the result from conventional radiographs is inconclusive and (2) when a middle third root fracture is seen in conventional radiographs (to assess oblique fractures). In our patient, CBCT was used because a new fracture was suspected from periapical radiographs. According to the additional findings revealed from CBCT, the importance of occlusal adjustment, avoidance of biting directly on the tooth, optimal oral hygiene practice, and periodic recalls were emphasized to the patient.

## 4. Conclusions

1. The location and healing patterns of horizontal root fractures seen on conventional radiographs and CBCT may be different.
2. Treatment of only the necrotic coronal root fragment of fracture by MTA obturation has a favorable outcome.
3. Long-term root canal medication with calcium hydroxide in teeth with horizontal root fracture was cautioned for the possibility of further fractures.
